# The effect of attention shifting on Chinese children’s word reading in primary school

**DOI:** 10.1186/s41155-024-00290-6

**Published:** 2024-02-27

**Authors:** Hui Zhou, Meiling Jiang

**Affiliations:** 1 Center for Brain, Mind and Education, Shaoxing University, Shaoxing City, People’s Republic of China; 2 Department of Psychology, School of Teacher Education, Shaoxing University, Shaoxing City, People’s Republic of China; 3 School of Teacher Education, Shaoxing University, Shaoxing City, People’s Republic of China

**Keywords:** Attention shifting, Word reading, Reading accuracy, Reading fluency, Primary school students

## Abstract

**Background:**

This study explored the effects of attention shifting on Chinese children’s word reading.

**Objective:**

The sample consisted of 87 fourth-grade children from Shaoxing City, China.

**Methods:**

The students completed measures of the attention shifting task, reading accuracy test, reading fluency test, and rapid automatized naming test.

**Results:**

The results showed that reading fluency was significantly correlated with attention shifting scores, specifically with tag1 and tag6 (*ps* < 0.05). The reading accuracy score was also significantly correlated with tag6 (*p* < 0.05). According to the regression analysis of attention shifting on word reading, even when controlling for rapid automatic naming, attention shifting significantly affected word reading fluency at approximately 600 ms (*p* = .011). Attention shifting did not affect children’s word reading accuracy.

**Short conclusion:**

These findings suggest that attention shifting is significantly associated with children’s word reading. Educators should focus on developing children’s attention shifting to improve their word reading ability.

## Introduction

Reading is important for children to obtain information and knowledge. Temporal attention affects the process of reading (Ruffino et al., 2014). When processing a continuous stream of stimuli, temporal attention can enhance limited cognitive resources and improve perceptual sensitivity within short time intervals (Denison et al., 2021). During the reading process, children need to shift their attention rapidly between reading materials, such as words. Attention contributes to continuous word scanning, which promotes successful reading (White et al., 2019). If children are unable to effectively control and allocate their temporal attention to reading, they exhibit poor reading proficiency (Dittman, 2016; Rezaei & Mousanezhad Jeddi, 2020).

### The relationship of attention shifting and children’s reading

Attention shifting refers to temporal attention allocation between multiple target stimuli, including attentional disengagement from the previous target stimulus and engagement with the next target stimulus (Facoetti et al., 2008). Previous research has shown that readers with poor reading abilities cannot shift their attention quickly from one stimulus to another and consistently perform worse on attention shifting tasks (Hari & Renvall, 2001; Vidyasagar & Pammer, 2010). It may take a long time to shift attention between two target stimuli. Hari et al. (1999) first employed the rapid serial visual presentation (RSVP) task, which is a dual task including a rapid series of stimuli (Tınok et al., 2023). The RSVP task is generally used to test temporal attention allocation. Hari et al. (1999) found that individuals who performed worse in reading also exhibited a longer attention shifting time (700 ms) in the RSVP task. The same relationship between reading and attention shifting has been shown in normally developing readers (McLean et al., 2009). Although the RSVP task is generally used to explore the phenomenon of attention blink, research has shown that in temporally multiple target processing, it is not attention blink (within 200–500 ms) but attention shifting that affects reading (Badcock et al., 2008; Zhang et al., 2022).

Although Hari et al. (1999) suggested that attention shifting affects reading ability in typically developing children, previous studies have mostly focused on children with reading difficulties. However, there is still insufficient evidence on the relationship between attention shifting and reading ability in typically developing children. Attention, especially temporal attention, is a powerful predictor of children’s reading abilities (de Diego-Balaguer et al., 2016; Larsen et al., 2022; Russo et al., 2021). In primary school, children learn to master multiple reading skills in order to recognize words (Acha et al., 2023; Torgesen et al., 1997). Children need to shift their attention quickly between reading skills for fluent reading (Kocaarslan, 2022; Schwanenflugel et al., 2004). Thus, attention shifting plays an important role in children’s word reading. Even under the control of reading skills, such as phonological awareness and rapid automatized naming, attention shifting still has a significant predictive effect on children’s word reading (de Groot et al., 2015).

### The effects of attention shifting on word reading accuracy and word reading fluency

As aforementioned, several studies have suggested a close relationship between attention shifting and children’s word reading. However, the effect of attention shifting on word reading remains unclear. Word reading ability is expressed in terms of accuracy and fluency. Word reading accuracy refers to the ability to recognize or decode words correctly (Hudson et al., 2005). Reading fluency is commonly defined as reading accurately and rapidly using appropriate prosody (Hudson et al., 2008). In primary school, children’s word reading develops quickly (Milankov et al., 2021; Monster et al., 2022), and attention inevitably participates in this process. Huang et al. (2019) examined the relationship of visual attention and fluent reading and found visual attention could significantly account for the variance in reading fluency among children in lower grades in primary school. Visual attention was a significant predictor of sentence-reading fluency among children in higher grades in primary schools. Valdois et al. (2019) proposed that visual perceptual processing has only a moderate impact on children’s reading. Word reading involves accurate letter processing, and visual attention enhances letter identification. In multi-element processing, children’s temporal visual attention predicted their reading fluency performance in primary school one year later. Thus, this study compared the effects of visual perceptual processing and temporal attention shifting on children’s word reading accuracy and fluency.

In addition to attention shifting, reading skills, especially rapid automatic naming (RAN), are important factors that significantly affect children’s word reading. RAN assesses children’s ability to rapidly name a series of materials such as letters, words, colors, or objects (Nadiyah et al., 2022; Norton & Wolf, 2012). It represents the speed of cognitive processing and has been widely recognized as a reliable predictor of children’s reading proficiency (Das & Samantaray, 2023; Savage et al., 2018). Layes et al. (2015) explored the relationship between cognitive reading skills and children’s word reading abilities in the fourth and fifth grades. They found that rapid naming and visual attention were significantly associated with reading ability. Compared to phonological processing, the relationship between RAN and word reading varies according to the reading material (Song et al., 2016). Therefore, RAN can affect the relationship between temporal attention and children’s word reading using different language materials. In the current study, we tested children’s RAN as a control factor to avoid confusion regarding the effects of attention shifting on children’s reading.

Previous studies have mostly examined the relationship of attention shifting and children’s word reading in alphabetic language (Franceschini et al., 2017; Meyer & Schaadt, 2020). However, the relationship between attention shifting and children’s reading ability may differ in different language systems. For example, attention shifting and emerging literacy skills were not significantly associated with French-speaking kindergarten children (Monette et al., 2011). Van der Sluis et al. (2007) found that the relationship between executive function and reading was considered negative in Dutch-speaking primary school students. Writing systems can be an important factor influencing reading mechanisms. Individuals speaking languages with different writing systems develop specific perceptual and cognitive mechanisms for efficient reading (Li et al., 2022). Unlike alphabetic languages, Chinese characters, as ideographic characters, are writing systems with deep orthographies (DeFrancis, 1989). Owing to the absence of a grapheme-to-phoneme correspondence rule for naming, Chinese characters can only be named through lexical or direct routes (Lee et al., 2004). Non-transparent orthography-to-phonology mapping may influence the rapid attention shift and RAN in children’s word-processing efficiency (Cheng et al., 2022; McMillen et al., 2020). The current research used a non-alphabetic language, which enriches research on the relationship between attention shifting and children’s word reading.

### The present study

Based on a literature analysis, we found that the effect of attention shifting on children’s word reading is still unclear, especially regarding word reading accuracy and word reading fluency. Therefore, this study aimed to explore the relationship between attention shifting and children’s word reading. Our hypotheses were as follows:

- Hypothesis 1: There is a positive correlation between attention shifting and word reading among primary school children.
- Hypothesis 2: Attention shifting significantly affects children’s word reading accuracy and fluency.

## Materials and methods

### Participants

In this study, 87 fourth-grade students were randomly selected from a primary school in Shaoxing City, Zhejiang Province, China. Shaoxing is a city in the central area of the Yangtze River Delta, where students can receive a good education. The students’ ages ranged from 9.3 to 11.9, with a mean age of 10.4 years (*SD* = 0.5). The participants comprised 44 boys (50.58%) and 43 girls (49.42%). According to the Raven’s test, no students had intellectual difficulties (scores < 70; Gonzalez et al., 2016; Zhang et al., 2011). Informed consent was obtained from the school teachers, parents of the students, and children. This study was approved by the Ethics Committee of Shaoxing University.

### Method

#### Attention shifting tasks

RSVP tasks were used to examine children’s attention shifting. The experiment was controlled by a PC running E-prime software. The stimuli were presented on a 17 inch cathode ray monitor. During the experiment, the students viewed a series of digits on a computer screen at a distance of 60 cm. The experiments were conducted in a quiet classroom. The children participated in the experiments independently during the activity classes. Before the experiment, the experimenters explained the tasks in detail to ensure that the children understood how to complete them.

**Fig. 1:**
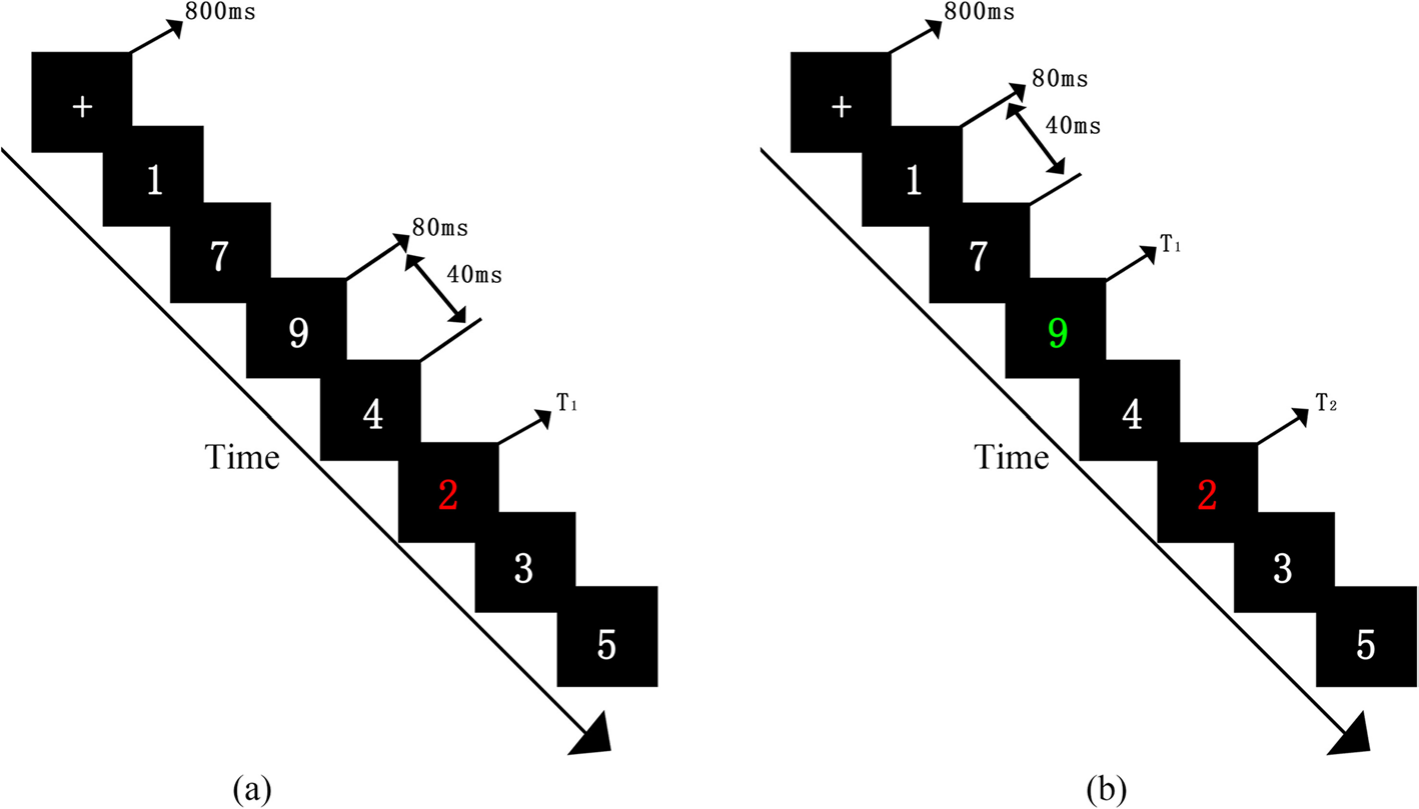
**a** An example of stimuli in a single trial during the single-target RSVP task. **b** An example of stimuli in a single trial during the dual-target RSVP task

In this study, the RSVP included single- and dual-target conditions. As shown in Fig. 1, in the single-target condition, a target digit (red digit) was presented in a series of no-target stimuli (white digits). Each trial began with a white fixation “ + ” in the center of a black screen (lasting 800 ms). After fixation, each digit was presented for 80 ms, followed by a black screen for 40 ms. After to 4–7 random white digits (non-target stimuli), the target digit was presented. At the end of each trial, the numbers 1–9 appeared in the center of the screen. Students were asked to choose the same number of digits they saw.

In the dual-target condition, the two target stimuli were presented in a series of no-target stimuli. The first target was a digit randomly selected from to 1–9 in red (T1). The second target was a digit that was randomly selected from to 1–9 in green (T2). There were one to seven no-target digits between T1 and T2. Therefore, there were six intertrial intervals (ITIs): 120, 240, 360, 480, 600, 720, and 840 ms (notated as tag1, tag2 ……tag7). As a control condition, the single-target task reflected the visual search ability of participants for a series of stimuli. The dual-target task reflected the participants’ attention shifting between two targets in a temporal series of stimuli. The experiment consisted of 10 practice tasks and 70 formal trials, which were presented randomly.

#### Reading accuracy test

The reading accuracy test used character recognition developed by Li et al. (2012). Owing to a large number of polyphone characters in Chinese, in order to avoid phonetic confusion, the reading accuracy test used two-word Chinese characters as materials, for example “看见.” The test included 220 two-word arrangements in order of increasing difficulty. The participants were asked to read the texts in sequence. If encountering words that they could not read, they could report “I don’t know.” If the participants read the two-word terms completely correctly, they obtained one point. However, if they read only one word incorrectly or did not recognize it, they received a score of zero. If a participant accumulated 15 zero points, the test was stopped. The reading accuracy score was the total number of words that the participants read correctly. The alpha coefficient for the reading accuracy test was 0.982.

#### Reading fluency test

The reading fluency test used a One-Minute Reading Task from HKT-SpLD (Ho et al., 2000). The test includes 150 high-frequency two-word Chinese characters (such as “白云”). The words mainly came from first-grade elementary school textbooks, which are relatively easy to read. This test required children to read words as quickly and accurately as possible within a one-minute time limit. If they encountered unfamiliar words, they could say “I don’t know.” Reading a two-word term correctly earned one point, whereas reading it incorrectly or not recognizing it earned zero points. The reading fluency score was the number of words read correctly in one minute. The alpha coefficient of the test was 0.976.

#### Rapid automatic naming test

The RAN test was adapted from the RAN/RAS test battery (Wolf & Denckla, 2005). The test includes three subtests: the digit RAN test, color RAN test, and words RAN test. Children were required to quickly and accurately name digits, colors, and high-frequency words sequentially. Each correct name earned a point. During the testing, a stopwatch recorded the time the participants read each time. Each subtest was tested twice and scored as the average of the two subtest times. The RAN test score is the average time of completion of the two subtests. In this study, the alpha coefficient was 0.846.

### Data analysis

This study used SPSS 26.0 for the data analysis, and the primary analysis methods included descriptive statistics, correlation, and regression analyses.

## Results

### Descriptive analysis with attention shifting, rapid naming, and reading ability

As shown in Table 1, the RAN score significantly correlated with the RSVP dual-target accuracy score, character recognition score, and one-minute reading score but not with the accuracy score of the RSVP single-target condition. The One-Minute Reading Task score was significantly correlated with the two-target accuracy score of the RSVP dual-target condition, specifically with tag1 and tag6 (*p*s < 0.05). The character recognition score was significantly correlated with tag6 (*p* < 0.05). The results suggested that children’s word reading ability was significantly correlated with the dual-target RSVP task, especially with tag6, indicating that attention shifting was significantly related to word reading at approximately 720 ms.

**Table 1:** Descriptive analysis with attention shifting, rapid naming and reading ability

	*Mean*	*SD*	1	2	3	4	5	6	7	8	9	10	11	12	13
1RAN	-.188	2.678	1												
2 RSVP single	26.167	1.924	-.219	1											
3 RSVP T1	64.677	4.386	-.418^**^	.507^**^	1										
4 RSVP T2	64.339	4.645	-.455^**^	.274^*^	.792^**^	1									
5tag1	8.129	1.624	-.362^**^	.121	.574^**^	.670^**^	1								
6tag2	8.935	1.099	-.273^*^	.149	.472^**^	.704^**^	.308^*^	1							
7tag3	9.242	.970	-.225	.098	.524^**^	.684^**^	.271^*^	.522^**^	1						
8tag4	9.565	.692	-.255^*^	.345^**^	.417^**^	.618^**^	.167	.372^**^	.282^*^	1					
9tag5	9.403	.914	-.380^**^	.084	.499^**^	.670^**^	.307^*^	.451^**^	.406^**^	.360^**^	1				
10tag6	9.500	.805	-.340^**^	.221	.562^**^	.713^**^	.401^**^	.445^**^	.305^*^	.544^**^	.368^**^	1			
11tag7	9.565	.842	-.251^*^	.397^**^	.618^**^	.600^**^	.198	.164	.452^**^	.541^**^	.317^*^	.423^**^	1		
12 One-minute reading	83.919	17.544	-.740^**^	.104	.323^*^	.284^*^	.273^*^	.103	.131	.220	.085	.277^*^	.215	1	
13 Character recognition	130.984	19.250	-.515^**^	.235	.237	.240	.225	.225	-.002	.127	.047	.276^*^	.180	.549^**^	1

*RAN* rapid automatic naming, *RSVP single* the single target condition of RSVP, *RSVP T1* T1 accuracy in the dual target condition of RSVP, *RSVP T2* T2 accuracy in the dual target condition of RSVP

^*^ *p < 0.05,* ^**^p < 0.01

### Regression analysis of attention shifting on word reading fluency

To examine the effect of attention shifting on word reading fluency, this study analyzed the effect of dual-target stimuli interval time in an attention shifting task on reading fluency. The results, shown in Table 2, indicated that tag5 still significantly affected reading fluency, even after controlling for RAN. This suggests that even when controlling for the effect of RAN on reading fluency, attention shifting significantly affected word reading fluency at approximately 600 ms.

**Table 2:** Regression analysis of attention shifting on word reading fluency

	*B*	*SE*	*Beta*	*t*	*p*
(Constant)	115.266	30.851		3.736	.000
RSVP single	-1.148	.899	-.125	-1.277	.208
tag1	.285	1.080	.027	.264	.793
tag2	-.579	1.956	-.036	-.296	.769
tag3	-.186	2.172	-.010	-.086	.932
tag4	1.036	3.253	.041	.318	.751
tag5	-5.397	2.050	-.282	-2.632	.011
tag6	2.394	2.677	.107	.894	.375
tag7	2.112	2.658	.102	.795	.431
RAN	-5.275	.649	-.810	-8.125	.000

### Regression analysis of attention shifting on word reading accuracy

To examine the effect of attention shifting on word reading accuracy, this study analyzed the interval effect of dual-target stimuli on reading accuracy in attention shifting tasks. The results, shown in Table 3, suggest that after controlling for RAN, there was no significant effect on reading accuracy for all tags. This suggests that attention shifting does not affect children’s word reading accuracy.

**Table 3:** Regression analysis of attention shifting on word reading accuracy

	*B*	*SE*	*Beta*	*t*	*p*
(Constant)	127.574	43.609		2.925	.005
RSVP single	.681	1.271	.068	.536	.595
tag1	.307	1.526	.026	.201b	.841
tag2	5.106	2.765	.291	1.847	.071
tag3	-5.920	3.070	-.298	-1.928	.060
tag4	-4.542	4.599	-.164	-.988	.328
tag5	-4.579	2.898	-.219	-1.580	.120
tag6	3.278	3.784	.134	.866	.390
tag7	4.863	3.757	.214	1.294	.202
RAN	-3.584	.918	-.503	-3.905	.000

## Discussion

In the current study, we found that reading fluency was significantly correlated with the RSVP dual-target accuracy score, specifically for tag1 and tag6. Reading accuracy was also significantly correlated with tag6, which suggested that attention shifting was significantly related to word reading at around 720 ms. Furthermore, according to the regression analysis of attention shifting on word reading, even controlled RAN, attention shifting significantly affected word reading fluency at around 600 ms. Attention shifting did not affect children’s word reading accuracy.

### Performance in dual-target but not single-target relates with children’s word reading

In this study, we found that only the dual-target task score was significantly correlated with word reading. The score on the single-target RSVP task did not correlate with the children’s word reading ability. The single- target RSVP task mainly reflects the perceptual processing ability of children during rapid processing of visual stimuli. These results were consistent with those of previous studies. Tyson-Parry et al. (2015) found that the single-target accuracy of the RSVP did not mediate the relationship between attention blink and reading in typical adults. Moreover, they found little difference in single-target accuracy between different groups in terms of reading ability. Several studies have suggested that perceptual processing, particularly visual processing, is partially a general basis for reading ability (Boden & Giaschi, 2007; Gabay et al., 2017; Whitford et al., 2023). Attention may expand the impact of perceptual processing on reading (Frey & Bosse, 2018; Stenneken et al., 2011). For example, when decoding a word, attention can implicitly direct perceptual processing toward the modality-specific perceptual information of a word, which, in turn, affects lexical decisions or reading responses (Connell & Lynott, 2014). Another possible explanation is that the cognitive load of dual-target tasks was higher than that of single-target tasks. Badcock et al. (2008) tested adults with dyslexia and found no difference between the dyslexia and control groups in a single-target RSVP task. They suggested that the cognitive load may be a problem for individuals with reading difficulties when completing dual tasks. A study of Chinese children found that although visual skills are significantly correlated with word recognition in younger children, the influence of visual skills on word reading disappears as children grow up (Li et al., 2012). This suggests that as the cognitive load of children’s reading decreases, perceptual processing does not affect their reading.

In this study, contrary to rapid visual processing, attention shifting was closely associated with reading in typically developing children. This suggests that attention shifting not only affects individuals with dyslexia but also plays an important role in children with typical development. According to the theory of sluggish attention shifting, Hari et al. (1999) proposed that compared with typical participants, individuals with dyslexia exhibited a significantly prolonged attention shifting time, and several studies have confirmed a delay in attention shifting among individuals with reading difficulties. For example, Visser et al. (2004) proposed that when processing sequential stimuli rapidly, children with developmental dyslexia show delayed attention allocation. These studies suggest that attention shifting is an important mechanism in reading processing. Reading a word involves several skills such as phonological awareness and decoding (Wood et al., 2023). When decoding words, children need to shift their attention rapidly between different processes such as phonological processing and orthography (Franceschini et al., 2017; Lan et al., 2023). In the middle stage of primary school, children have mastered separate reading skills but are not yet proficient in integrating reading skills for word decoding (Landerl & Wimmer, 2008; Milankov et al., 2021). Therefore, attention shifting plays a particularly important role in children’s reading processing during the middle stages of primary school.

### Attention shifting affects children’s word reading fluency but not reading accuracy

This study found that attention shifting was significantly related to word reading. Further regression analysis showed that attention shifting significantly affected word reading fluency at approximately 600 ms, even when controlling for RAN. Attention shifting did not affect children’s word reading accuracy. These results were consistent with those of previous studies. Several studies have proposed that temporal attention affects children’s reading (de Diego-Balaguer et al., 2016; Taran et al., 2022). For example, de Groot et al. (2015) employed the RSVP task test with Dutch school children in Grades 3–6. They found that children’s performance on the RSVP predicted their word reading and controlled phonemic awareness and alphanumeric rapid naming. However, previous studies have not explored whether attention shifting affects word reading accuracy, fluency, or both. This study confirmed that attention shifting only affects children’s word reading fluency.

One possible explanation is that reading fluency involves multiple processes in a short period; therefore, temporal attention shifting plays a greater role in reading fluency. When recognizing words, children not only need to decode words accurately but also require a quick rate with appropriate prosody (Hudson et al., 2008). Reynolds and Besner (2006) suggested that attention plays an important role in reading fluency, especially in transforming written material into spoken language. Yildiz and Çetinkaya (2017) tested fourth-grade children and found attention more closely related with reading speed than word recognition and comprehension. They proposed that word recognition is a basic reading process for good readers. If children master reading skills, they can decode words quickly and pay more attention to voice, intonation, and so on. Several studies have confirmed that temporal attention can affect children’s inefficient wording decoding (Arrington et al., 2014; Driver, 2001; Li et al., 2023).

Another possible explanation involves the processing characteristics of Chinese word reading. Chinese characters are presented as square boxes and are composed of one or more radicals with strokes (Li et al., 2022). Yin et al. (2023) have suggested that Chinese word recognition involves complex cognitive and linguistic processes. In Chinese word decoding, attention is closely related to orthographic mapping. Unlike proficient readers in fast orthographic-phonological mapping, early learning to read involves both phonological and orthographic skill training (Lin & Zhang, 2023). Therefore, temporal attention plays an important role when inexperienced readers read quickly. For example, Huang et al. (2019) found a close relationship between visual attention and fluent reading in Chinese word reading.

In this study, we further confirmed that attention shifting in the temporal process affects children’s word reading fluency. An important time point at which it does so is approximately 600 ms. Several studies have employed RSVP tasks to examine the relationship between attention shifting and reading. They have suggested that delayed attention shifting, which occurs later than the attention blink time, leads to poor reading performance (e.g., Badcock et al., 2008; Badcock & Kidd, 2015). Meyer and Schaadt (2020) examined third- and fourth-grade children in Germany and found attention shifting deficit in children with reading problems. They suggested that the delayed shifting of attention in rapid temporal processing may be due to the extension of the perceptual processing time window, resulting in a long-time sampling input. After the perceptual processing of target stimuli, children who are not proficient in word reading or have difficulty are affected more by attention shifting.

Therefore, teachers and parents should focus on shifting children’s attention during the middle stage of primary school. In the early stages of primary school, parents and teachers often pay more attention to training children’s reading skills. However, they often overlook the integration of multiple reading skills and attention roles in word reading. According to the simple reading view, middle grade in primary school is the key period for children to be able to read fluently. The current research suggests that attention shifting training may improve children’s word reading performance, especially fluent word reading.

### Limitations and implications

The present study has several limitations. First, in addition to word reading, children’s multiple reading skills and reading comprehension develop rapidly in primary school. Further research should focus on the effect of attention shifting on children’s reading abilities. Second, this study only recruited participants from a primary school in Shaoxing City. Therefore, the sample size and subject representativeness were limited. In future research, subjects from different places should be recruited to increase their diversity and representativeness. Third, this study did not include children’s academic achievement scores. Children with reading difficulties may also experience other learning difficulties. Therefore, in the current study, children’s reading performances may have been confused with learning factors. Finally, we only adopted a cross-sectional design, which cannot explain the developmental relationship between attention shifting and word reading. Future research should further track the developmental relationships between childhood variables.

## Conclusion

Attention shifting was significantly related to children’s word reading. Even with controlled RAN, attention shifting significantly affected word reading fluency at approximately 600 ms. Attention shifting did not affect the children’s word reading accuracy.

## Data Availability

The datasets generated and/or analyzed in the current study are available from the corresponding author upon reasonable request.

## Abbreviations

RAN: Rapid automatized naming
RSVP: Rapid serial visual presentation
